# Prognostic factors in metastatic pancreatic cancer: Older patients are associated with reduced overall survival

**DOI:** 10.3892/mco.2013.131

**Published:** 2013-05-23

**Authors:** FARUK TAS, FATMA SEN, SERKAN KESKIN, LEYLA KILIC, IBRAHIM YILDIZ

**Affiliations:** Institute of Oncology, University of Istanbul, 34390 Istanbul, Turkey

**Keywords:** pancreatic cancer, metastatic, elderly, prognostic factor, survival

## Abstract

Pancreatic cancer is a major health concern worldwide and, despite the attempts at management, the prognosis of patients with metastatic pancreatic cancer remains poor, with a median survival of a few months. The aim of this study was to identify and evaluate clinicopathological factors and elucidate the clinical significance of patient age for the outcome of metastatic pancreatic cancer. Data from a total of 154 metastatic patients with a histologically confirmed diagnosis of pancreatic cancer, who were treated and followed-up in our clinic, were recorded from medical charts. The patient sample included 102 (66%) males with a median age of 58 years (range, 25–88 years). The majority of the patients had a poor performance status (64%), weight loss of >10% body weight (74%), tumor size of >3 cm (75%) and elevated tumor markers, including carcinoembryonic antigen (CEA) (66%) and carbohydrate antigen 19-9 (CA19-9) (85%). The distributions of prognostic factors depending on patient age were largely identical. The median survival time of patients with metastatic disease was 179 days and the 1-year survival rate was 7%. The median survival time of elderly patients was significantly lower compared to younger patients (148 vs. 198 days, respectively; P=0.039). The 1-year survival rates in elderly and younger patients were 3 and 10%, respectively. In the univariate and multivariate analyses, elderly patients had poorer outcomes compared with younger patients (P=0.04 and 0.05, respectively). In all patients, the univariate analysis demonstrated that similar prognostic factors, including the performance status of the patients and tumor markers, such as serum CEA and CA19-9 levels, were associated with survival. In the multivariate analysis, younger patients with a poor performance status had a significantly shorter overall survival compared to those with a good performance status (P=0.008). However, no significant prognostic factor affecting the outcome was identified in the elderly patients. In conclusion, patient age is a major prognostic factor affecting the survival of patients with metastatic pancreatic cancer and elderly patients without poor prognostic factors may be eligible for the available treatment options.

## Introduction

Pancreatic cancer is a major health problem with a poor prognosis. In the USA it is the fourth leading cause of cancer-related mortality in both genders (1) and as many as 55% of patients are diagnosed at a metastatic stage. Despite the attempts at management, prognosis of metastatic patients is poor, with a median survival of ∼3–6 months and a 5-year survival rate of 2% (1).

Non-surgical treatment options, such as chemotherapy or targeted therapy, have been investigated with regard to whether they prolong the overall survival of patients with metastatic pancreatic cancer. Due to the moderate improvement achieved by chemotherapeutics, recent studies evaluated whether subgroups of patients may be identified who would benefit the most from specific treatment strategies (2–4). This may improve the identification of patients with a poor prognosis and subsequent administration of supportive care alone, which may help avoid the unnecessary adverse effects and complications of systemic chemotherapy.

Pancreatic cancer is a disease that mainly affects the elderly population (2). The incidence of pancreatic cancer increases with age, with 60% of patients >65 years (2–5). Patient age has been a well-recognized prognostic factor in numerous types of cancer treated with definitive intent. In addition to the pretreatment serum hemoglobin levels, initial serum carbohydrate antigen 19-9 (CA19-9), carcinoembryonic antigen (CEA) and lactate dehydrogenase (LDH) levels have been identified as significant prognostic factors in different stages of pancreatic cancer. Furthermore, several studies have demonstrated that patient age is an important independent prognostic factor affecting survival (6,7). Moreover, elderly patients usually benefit from single and/or combination chemotherapy regimens.

In a previous study, we investigated the immediate and long-term outcome in a limited number of patients with pancreatic cancer and evaluated the possible impact of different clinicopathological factors on survival (8). The aim of this study was to identify and evaluate the same clinicopathological factors in a larger cohort and elucidate the clinical significance of patient age for the outcome of metastatic pancreatic cancer.

## Materials and methods

### Patients

Data from 154 patients with histologically confirmed diagnosis of metastatic pancreatic cancer, treated and followed-up in our clinic, were recorded from medical charts. Tumor localization was determined surgically, endoscopically or radiologically and pathological confirmation of pancreatic cancer was obtained by surgery or by fine-needle aspiration biopsy. Staging of metastatic patients was performed with various imaging modalities, such as computed tomography (CT), magnetic resonance imaging and positron emission tomography (PET)/CT scan. Patients were staged according to the International Union Against Cancer TNM classification. Written informed consent was obtained from all patients for their participation in this study. This study was approved by the Institute of Oncology, University of Istanbul (Istanbul, Turkey).

### Treatment and prognostic variables

Chemotherapy was administered to the majority of the patients with metastatic disease (n=113, 73%). Patients with metastatic disease were treated with various single-agent or combination chemotherapeutic regimens, selected according to the performance status of the patients and the extent of the disease. Drug schemes were applied as follows: gemcitabine alone, combination of gemcitabine with platinum, capecitabine alone or fluorouracil (5-FU) with folinic acid. Response to chemotherapy was evaluated radiologically after 2–3 cycles of chemotherapy according to international criteria. Patients not responding to chemotherapy were treated with second-line chemotherapy, provided they had a good performance status. Chemotherapy was continued until disease progression or unacceptable toxicity.

The possible prognostic variables were selected based on those identified in previous studies (6–8). Serum CEA and CA19-9 levels were determined by microparticle enzyme immunoassay (Abbott Diagnostics, Chicago, IL, USA). Serum LDH, albumin and hemoglobin levels were measured at presentation in our biochemical laboratory. Serum LDH activity was determined immediately after collection by the kinetic method on a Targa-3000 autoanalyzer (Pointe Scientific Inc., Lincoln Park, MI, USA) at 37°C. The laboratory parameters were evaluated at diagnosis within the normal ranges of our institition.

### Statistical analysis

SPSS software version 16 (SPSS, Inc., Chicago, IL, USA) was used for statistical analysis. Quantitative analysis was summarized by median, minimum and maximum and qualitative analyses were presented as frequencies and percentages. The Chi-square test was used to assess the differences in the distribution of the clinicopathological parameters of the metastatic disease. Overall survival was determined as the time elapsed between the time of histological diagnosis and the date of death, the date of the last follow-up visit or the point of the study at which the patient was still alive. Time dependent variables and overall survival were estimated with the Kaplan Meier method and their differences were evaluated by the log-rank test. Multivariate analysis (Cox proportional hazards model) was used to determine the variables with an independent effect on survival. All deaths were considered as events, regardless of their cause. P≤0.05 was considered to indicate a statistically significant difference.

## Results

### Clinicopathological characteristics

The demographic, laboratory and clinicopathological characteristics of the patients are listed in Table I. In this retrospective study, the outcome of 154 patients with metastatic pancreatic cancer treated and followed-up in our clinic was analyzed. Of these, 102 (66%) were male, with a median age of 58 years (range, 25–88 years). The majority of the patients had a poor performance status (64%), weight loss >10% body weight (74%), tumor size of >3 cm (75%) and elevated tumor markers, including CEA (66%) and CA19-9 (85%). Moreover, the rate of response to chemoterapy was 24%.

The distributions of prognostic factors depending on patient age were generally identical (Table I). Specifically, the percentage of patients with larger tumors was higher among elderly compared to younger patients (64 vs. 90%, respectively; P=0.002). However, fewer elderly patients were anemic compared to younger patients (28 vs. 47%, respectively; P=0.048).

### Overall survival

**Figure 1.**
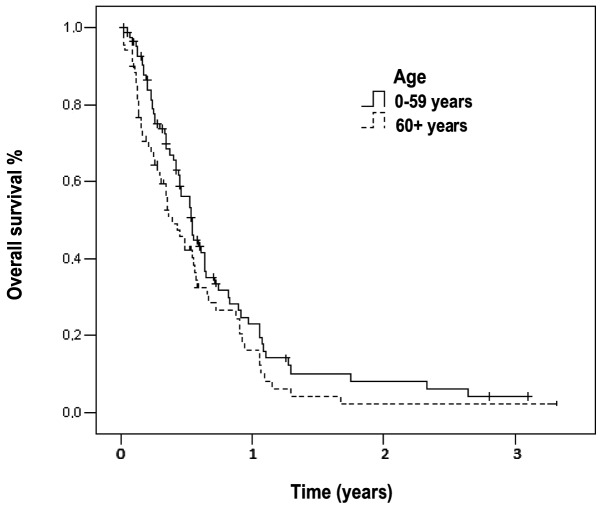
Overall survival of patients with metastatic pancreatic cancer according to patient age (P=0.039).

The median follow-up time was 290 days (range, 1–78 months) for all the patients. At the time of the analysis, only 32 (21%) patients were alive. The median survival time of patients with metastatic disease was 179 days (95% CI: 148–209) and the 1-year survival rate was 7% (Fig. 1). In the subset analysis, we noted that out of the 32 surviving patients, 12 (17%) were elderly and the remaining 20 (24%) belonged to the younger age group. The median survival time of the elderly patients (144 days, 95% CI: 90–197) was significantly lower compared to that of younger patients (198 days, 95% CI: 165–230, P=0.039). The 1-year survival rates for elderly and younger patients were 3 and 10%, respectively (Fig. 1).

In the univariate analysis, elderly patients had poorer outcomes compared to younger patients (median survival, 114 vs. 198 days, respectively; P=0.04) (Table II). In addition, patients with a poor performance status, high CA19-9 and CEA levels, jaundice, leukocytosis and unresponsiveness to chemotherapy exhibited shorter survival. In the multivariate analysis, similar to other prognostic factors identified as significantly different in the univariate analysis, a significant difference was observed in elderly patients (P=0.048).

### Clinical significance of patient age

Table III summarizes the analysis of the association of patient age with various clinical and laboratory parameters. In both the elderly and younger patient groups, the univariate analysis identified the same prognostic factors, such as patient performance status and tumor markers, including serum CEA and CA19-9 levels, to be associated with survival. In the multivariate analysis, younger patients with a poor performance status had a significantly shorter overall survival compared to those with a good performance status (P=0.008). However, no significant prognostic factor affecting outcome was identified in the elderly patients.

## Discussion

It has been demonstrated that elderly patients are underrepresented in cancer clinical trials (2). Despite the fact that elderly patients account for the majority of cancer patients, they are markedly underrepresented in cancer clinical treatment trials, constituting only 30–40% of the number of cancer patients (9). In National Cancer Institute-sponsored clinical trials, <1% of adults 75–79 years of age are enrolled (4,10). Possible explanations for this are the presence of comorbidities and the limited expectations for long-term benefit of chemotherapy. For these reasons, exclusion of older individuals is considered acceptable based on non-eligibility criteria (9). Similarly, the treatment of elderly pancreatic cancer patients poses a significant challenge (4).

A recent study conducted on elderly patients with various malignancies demonstrated that elderly patients benefit from chemotherapy to a similar extent as younger patients, with manageable side effects (4). A meta-analysis of three randomized metastatic colon cancer studies, comparing combination chemotherapy with 5-FU compared to 5-FU monotherapy, demonstrated that patients >70 years of age benefited from the treatment similar to younger patients, with no apparent greater toxicity (11). Furthermore, gemcitabine is well-tolerated in elderly patients with other types of tumors (12).

Chemotherapy is as feasible in the elderly as in the younger pancreatic cancer patients (2,4,9). Recent studies suggested that the safety and efficacy of gemcitabine-based chemotherapy in elderly patients is similar to that in younger patients (2,9). The response rate and outcome were similar in elderly and younger patients (2). Furthermore, although the majority of very elderly patients (aged >80 years) with metastatic pancreatic cancer do not receive any treatment, the administration of chemotherapy in this particular patient population was associated with improved survival (4).

Age as a prognostic factor has been investigated in numerous studies with controversial results. A previous study described age as an independent prognostic factor (6) whereas others did not identify age as such a factor (2,9). The majority of studies on prognostic factors are questionable in terms of sample size and statistical methods, being largely based on small retrospective analyses (7). Data consisting a total of 34 possible prognostic factors from 653 advanced pancreatic cancer patients were analyzed and the log-rank analysis indicated that age was a potentially important factor affecting survival (7). In our study, we observed that age appears to affect survival. This result is consistent with those found in the literature.

Patients of identical age may greatly differ in their functional status (4). The performance status, which provides a useful guide in making treatment decisions for younger patients, is often insufficient to assess the overall status of elderly patients (4). The performance status is an independent negative prognostic factor for elderly patients (9).

In conclusion, almost all of the patients with metastatic pancreatic cancer have a poor prognosis and establishing definitive prognostic variables during the initial diagnosis may help physicians determine which patients should be considered for supportive care alone, single-agent chemotherapy, combination chemotherapy or multimodality treatment options. In this study, we demonstrated that patient age is a major prognostic factor affecting survival in metastatic pancreatic cancer. Age should not preclude patients from receiving chemotherapy and treatment decisions should be based on the physiological rather than the chronological age (4). The evaluation of factors including functional status, co-morbidity and cognition in elderly patients is necessary (4).

Therefore, elderly patients may be eligible for treatment options, provided they exhibit no weight loss, have a good performance status and favorable prognostic factors, such as normal tumor marker serum levels.

## Figures and Tables

**Table I.** Patient characteristics and distribution of prognostic factors based on patient age.

Characteristics	Poor prognostic factor	Patients			P-value
		Total (%)	Younger (<60 years, %)	Older (>60 years, %)	
Gender	Male	66	67	65	0.810
Performance status (ECOG)	Poor (2–4)	64	71	60	0.157
Weight loss	Present (>10% BW)	74	72	76	0.640
Jaundice	Present	42	43	40	0.830
Tumor location	Head	50	45	56	0.240
Tumor diameter	Large (>3 cm)	75	64	90	0.002
Hemoglobin levels	Anemia (<12 g/dl)	38	47	28	0.048
WBC count	Elevated (>10000)	31	31	31	0.990
Platelet count	Elevated (>450000)	10	16	2	0.170
Albumin levels	Low (<4 g/dl)	54	55	52	0.880
LDH levels	Elevated (>450 U/l)	29	24	36	0.300
CEA levels	Elevated (>4 ng/ml)	66	62	71	0.400
CA19-9 levels	Elevated (>37 IU/ml)	85	86	83	0.770
Response to chemotherapy	Absent (stable/progression)	24	24	24	0.870

ECOG, Eastern Cooperative Oncology Group; WBC, white blood cell; LDH, lactate dehydrogenase; CEA, carcinoembryonic antigen; CA, carbohydrate antigen; BW, body weight.

**Table II.** Prognostic factors predicting overall survival in patients with metastatic pancreatic cancer.

Factor	P-value	
	Univariate analysis	Multivariate analysis
Performance status (good vs. poor)	<0.001	0.002
CA19-9 level (normal vs. high)	<0.001	0.034
CEA level (normal vs. high)	0.001	0.034
Jaundice (present vs. absent)	0.004	0.043
Response to chemotherapy (present vs. absent)	0.030	0.047
WBC count (normal vs. high)	0.035	-
Age (older vs. younger)	0.040	0.048
Tumor location (head vs. others)	-	0.019

WBC, white blood cell; CA, carbohydrate antigen; CEA, carcinoembryonic antigen.

**Table III.** Prognostic variables for survival according to patient age.

Patients	Univariate analysis	P-value	Multivariate analysis	P-value
Younger (<60 years)	Performance status (poor vs. good)	<0.001	Performance status (poor vs. good)	0.008
	CA19-9 (normal vs. high)	0.003		
	Jaundice (present vs. absent)	0.014		
	CEA (normal vs. high)	0.018		
	Tumor location (head vs. others)	0.034		
Older (>60 years)	Performance status (poor vs. good)	<0.001	No independent risk factor	
	CA19-9 (normal vs. high)	0.033		
	CEA (normal vs. high)	0.041		
	Response to chemotherapy (present vs. absent)	0.049		
	Gender (female vs. male)	0.052		

CA, carbohydrate antigen; CEA, carcinoembryonic antigen.
